# The Influence of Tetrodotoxin (TTX) on the Distribution and Chemical Coding of Caudal Mesenteric Ganglion (CaMG) Neurons Supplying the Porcine Urinary Bladder

**DOI:** 10.3390/md15040101

**Published:** 2017-03-30

**Authors:** Ewa Lepiarczyk, Agnieszka Bossowska, Jerzy Kaleczyc, Marta Majewska, Sławomir Gonkowski, Mariusz Majewski

**Affiliations:** 1 Department of Human Physiology, Faculty of Medical Sciences, University of Warmia and Mazury in Olsztyn, 10-082 Olsztyn, Poland; agnboss@uwm.edu.pl (A.B.); marta.majewska@uwm.edu.pl (M.M.); mariusz.majewski@uwm.edu.pl (M.M.); 2 Department of Animal Anatomy, Faculty of Veterinary Medicine, University of Warmia and Mazury in Olsztyn, 10-719 Olsztyn, Poland; jerzy.kaleczyc@uwm.edu.pl; 3 Department of Clinical Physiology, Faculty of Veterinary Medicine, University of Warmia and Mazury in Olsztyn,10-719 Olsztyn, Poland; slawomir.gonkowski@uwm.edu.pl

**Keywords:** tetrodotoxin, caudal mesenteric ganglion, urinary bladder, immunohistochemistry, pig

## Abstract

The treatment of micturition disorders creates a serious problem for urologists. Recently, new therapeutic agents, such as neurotoxins, are being considered for the therapy of urological patients. The present study investigated the chemical coding of caudal mesenteric ganglion (CaMG) neurons supplying the porcine urinary bladder after intravesical instillation of tetrodotoxin (TTX). The CaMG neurons were visualized with retrograde tracer Fast blue (FB) and their chemical profile was disclosed with double-labeling immunohistochemistry using antibodies against tyrosine hydroxylase (TH), neuropeptide Y (NPY), vasoactive intestinal polypeptide (VIP), somatostatin (SOM), calbindin (CB), galanin (GAL) and neuronal nitric oxide synthase (nNOS). It was found that in both the control (*n* = 6) and TTX-treated pigs (*n* = 6), the vast majority (92.6% ± 3.4% and 88.8% ± 2%, respectively) of FB-positive (FB+) nerve cells were TH+. TTX instillation caused a decrease in the number of FB+/TH+ neurons immunopositive to NPY (88.9% ± 5.3% in the control animals vs. 10.6% ± 5.3% in TTX-treated pigs) or VIP (1.7% ± 0.6% vs. 0%), and an increase in the number of FB+/TH+ neurons immunoreactive to SOM (8.8% ± 1.6% vs. 39% ± 12.8%), CB (1.8% ± 0.7% vs. 12.6% ± 2.7%), GAL (1.7% ± 0.8% vs. 10.9% ± 2.6%) or nNOS (0% vs. 1.1% ± 0.3%). The present study is the first to suggest that TTX modifies the chemical coding of CaMG neurons supplying the porcine urinary bladder.

## 1. Introduction

Tetrodotoxin (TTX) is a toxic substance that may be found in some aquatic organisms, including the most famous pufferfish belonging to the family Tetraodontidae (for review see: [1]), as well as in several terrestrial species of newts, toads and frogs [2,3,4,5]. It is believed that the toxin is not produced by the animal organism itself, but by particular species of bacteria belonging to the genus *Vibrio*, which seem to enter their host’s body via the food chain. Therefore, the concentration of TTX in different organs varies depending on the concentration of the bacteria in certain tissues. In the pufferfish the most toxic organs are usually the ovary and liver, but there are some species in which TTX accumulates in the skin, intestines or muscles [6,7]. The main mode of TTX action involves blocking the voltage-gated sodium channels. This mode of action leads to a reduction in the membrane excitability of nerve fibers and skeletal or cardiac myocytes [8,9]. Since voltage-gated sodium channels play a key role in nociceptive transmission, attempts were undertaken to employ TTX in the treatment of pathological conditions associated with severe pain, such as rheumatoid arthritis, migraines and the terminal stage of cancer [10,11,12]. The use of this toxin has been also considered for medical purposes other than pain management, and appropriate experiments have been carried out in rats to treat, for example, corneal injury–induced photophobia [13] or schizophrenia [14]. Moreover, clinical trials on humans show that TTX is efficient in alleviating acute heroin withdrawal symptoms with few side effects [15].

The treatment of micturition disorders, especially those associated with over-activity of the urinary bladder, creates a serious problem for urologists. Although antimuscarinic drugs still remain the first choice in therapy, their use is limited by many side effects [16]. Therefore, new, more selective therapeutic agents, such as neurotoxins, are being investigated for the treatment of urological patients. Currently, there are several examples of potent natural toxins applied in human urology, including the most famous botulinum toxin (BTX) and resiniferatoxin (RTX). Both these toxins are used to treat lower urinary tract symptoms, particularly those associated with neurogenic detrusor overactivity, idiopathic detrusor overactivity or urgency (for recent reviews: [17,18,19]). Taking into account the beneficial therapeutic effects of intravesically instilled BTX or RTX, in the last few years the studies were expanded to include some other neurotoxins to the neurological resources. RTX acts by blocking the transient receptor potential 1 (TRPV1) cation channel (which is a nonspecific Ca^2+^ channel, previously known as the vanilloid receptor) of neurons involved in nociceptive signaling [20]. Therefore, the justification for RTX application in patients suffering from lower urinary tract symptoms is based on the ability of the neurotoxin to desensitize bladder C-fibers, and therefore to impair the excessive C-fiber input-dependent sacral micturition reflex [21]. As was previously mentioned, TTX, by blocking voltage-gated sodium channels, is also able to impair nociceptive transmission. The recognition of this capacity of the toxin has implied that it has been frequently used in experimental studies investigating physiological and pathological processes involved in mammalian urinary bladder function (e.g., [22,23,24]). Thus, the introduction of TTX, as an alternative to RTX, for the treatment of urological disorders will certainly be taken into account.

Studies dealing with the impact of neurotoxins on the chemical coding of neurons supplying the urinary bladder may enhance our understanding of the consequences of their action on the tissues treated. Our previous investigations have revealed that intravesical instillation of BTX or RTX results in profound changes in the chemical features of neurons in porcine sympathetic chain ganglia [25,26] or caudal mesenteric ganglia (CaMG) [27]. Bearing in mind that the general TTX therapeutic effect can be considered similar to that of RTX, the purpose of the present study was to investigate, by the use of combined retrograde tracing and immunohistochemistry, the putative influence of TTX intravesical instillation on the distribution and chemical coding of CaMG neurons projecting to the urinary bladder wall. We decided to use domestic pigs in the experiment as they share similar anatomic and physiologic characteristics involving the urinary, cardiovascular, integumentary and digestive systems with humans, and thus are considered to be one of the major animal species used in biomedical research (e.g., [28,29,30]).

## 2. Results

### 2.1. Distribution of Fast Blue-Positive (FB+) Neurons in the Control Pigs

FB+ neurons were found in the CaMG in all the pigs. The neurons were located within the left and right ganglia, with a distinct predominance in the ipsilateral ones. In the control animals (after the injection of FB into the right side of the urinary bladder wall) there were 722.5 ± 70.7 (93.3% ± 3.2%) dye-labeled neurons in the right and 54 ± 28.1 (6.7% ± 3.2%) in the left ganglion. Two subpopulations of ganglionic neurons were distinguished in the control animals: one consisted of large neurons (40–60 µm) and another one comprised smaller neurons (15–35 µm). In the right CaMG, there were 70.4% ± 8.3% of small and 29.6% ± 8.3% of large FB+ neurons. In the left CaMG, these values amounted to 70.2% ± 7% and 29.8% ± 7%, respectively.

In the ipsilateral ganglion, the majority of the urinary bladder-projecting neurons (UBPN) were located caudally, along the lateral ganglionic border. Additionally, a small group of the neurons was regularly found closer to the middle part of the ganglion. In the contralateral ganglion, the majority of FB+ nerve cell bodies was also located caudally, however they were much more dispersed and less numerous than those within the ipsilateral one.

### 2.2. Distribution of FB+ Neurons in the TTX-Treated Pigs

FB+ neurons were found in the CaMG in all the pigs. The neurons were located within left and right ganglia, with a distinct predominance in the ipsilateral ones. In the TTX-treated animals (after the injection of FB into the right side of the urinary bladder wall) there were 757.3 ± 113.6 (90.1% ± 8.1%) dye-labeled neurons in the right and 80.4 ± 63.4 (9.9% ± 8.1%) in the left ganglion. In the right CaMG, there were 70% ± 8.2% of small and 30% ± 8.2% of large FB+ neurons. In the left CaMG, these values amounted to 71.8% ± 6% and 28.2% ± 6%, respectively.

The distribution of the FB+ UBPN in the TTX-treated animals in both the ipsilateral and contralateral ganglia was similar to that observed in the control animals.

### 2.3. Immunohistochemical Characteristics of FB+ Neurons in the Control Pigs

**Figure 1:**
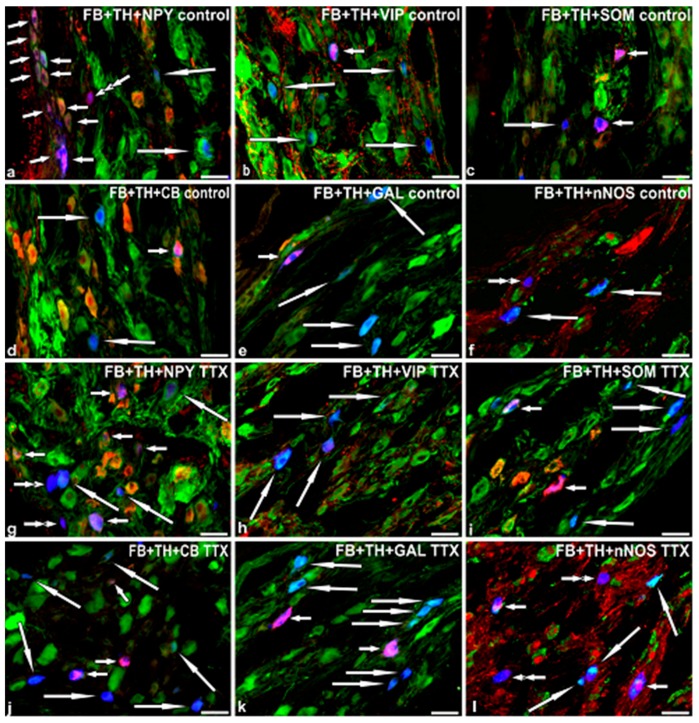
Representative images of caudal mesenteric ganglion urinary bladder-projecting neurons (CaMG-UBPN) in the control pigs (**a**–**f**) or in the TTX-treated pigs (**g**–**l**). All the images were combined from images taken separately from blue, green and red fluorescent channels. Blue fluorescent channel was used to visualize Fast blue positive (FB+) neurons (**a**–**l**). Green channel was used to visualize TH+ neurons (**a**–**l**). Red channel was used to visualize NPY+ (**a**,**g**), VIP+ (**b**,**h**), SOM+ (**c**,**i**), CB+ (**d**,**j**), GAL+ (**e**,**k**) or nNOS+ (**f**,**l**) nerve cells. Short arrows represent FB+ CaMG-UBPN which were simultaneously TH+ and NPY+ (**a**,**g**), VIP+ (**b**), SOM+ (**c**,**i**), CB+ (**d**,**j**), GAL+ (**e**,**k**) or nNOS+ (**l**). Long arrows represent FB+ CaMG UBPN which were simultaneously TH+ but NPY-negative (NPY−; **a**,**g**), VIP− (**b**,**h**), SOM− (**c**,**i**), CB− (**d**,**j**), GAL− (**e**,**k**) or nNOS− (**f**,**l**). Double arrows represent FB+ CaMG UBPN which were simultaneously both TH− and NPY− (**g**) or nNOS− (**f**,**l**). Triple arrow represents FB+ CaMG urinary bladder-projecting neuron which was simultaneously TH− but NPY+ (**a**). Bar in all images = 50 µm.

Double-labeling immunohistochemistry revealed two main populations of FB+ neurons. The vast majority of the retrograde-labeled nerve cells were tyrosine hydroxylase -positive (TH+; Table 1; Figure 1a–f). The remaining FB+ neurons were TH-negative (TH−; Table 2; Figure 1a,f).

Many FB+/TH+ neurons stained also for neuropeptide Y (NPY; Figure 1a). A small number of FB+/TH+ neurons were immunopositive to vasoactive intestinal polypeptide (VIP; Figure 1b), somatostatin (SOM; Figure 1c), calbindin (CB; Figure 1d) or galanin (GAL; Figure 1e). No FB+/TH+ nerve cells displayed immunoreactivity to neuronal nitric oxide synthase (nNOS; Figure 1f).

Some FB+/TH− (non-adrenergic) neurons simultaneously stained for NPY (Figure 1a). A small number of FB+/TH− neurons stained also for VIP. No FB+/TH− nerve cells containing immunoreactivity to SOM, CB, GAL or nNOS were encountered.

### 2.4. Immunohistochemical Characteristics of FB+ Neurons in TTX-Treated Animals

Double-labeling immunohistochemistry revealed that, similar to the control group, the vast majority of the retrograde-labeled nerve cells were TH+ (Table 1; Figure 1g–l). The remaining FB+ neurons were TH− (Table 2; Figure 1g,l).

However, in TTX-treated pigs the percentages of FB+ noradrenergic neuronal subpopulations distinctly differed from those determined in the control animals (Table 1).

A lower number of FB+/TH+/NPY+ neurons was observed in TTX-treated pigs (Figure 1g). Furthermore, in the toxin-treated animals no FB+/TH+/VIP+ nerve cells were observed (Figure 1h). On the other hand, TTX intravesical instillation resulted in a significant increase in the number of FB+/TH+ neurons immunopositive to SOM (Figure 1i), CB (Figure 1j), GAL (Figure 1k) or nNOS (Figure 1l).

In the TTX-treated pigs, the immunohistochemical coding of FB+/TH− neurons also differed from that found in the control animals (Table 2).

The changes consisted in the lower number of the perikarya stained for NPY and the absence of the neuronal somata containing immunoreactivity to VIP determined in the toxin-treated animals. On the other hand, TTX treatment was followed by an increase in the number of FB+/TH− nerve cells immunopositive to SOM, CB, GAL or nNOS.

## 3. Discussion

The present study provides further confirmation that CaMG plays a significant role in the autonomic supply of the urinary bladder in the female pig. The thorough discussion regarding the contribution of CaMG to the innervation of the female porcine urinary bladder has been already presented in our previous paper [27]. In both studies we used different control groups. In the earlier study [27], the control pigs were intravesically instilled with 5% aqueous solution of ethyl alcohol. In the present experiment, the intact animals were instilled with citrate buffer. The above-mentioned procedures were performed in the control pigs to ensure that changes in the chemical coding of neurons after the toxin treatment (RTX or TTX in the previous or the present study, respectively) were caused by the toxins themselves, not due to factors associated with the method of their application. Nevertheless, it must be stressed that the number, sex, body weight and age of the animals used as controls in both experiments as well as the surgical and immunohistochemical procedures applied were entirely corresponding. No statistically significant changes in the number of FB+ CaMG-UBPN or in the percentages of the immunopositive neurons were found between the control animals used in both studies. Therefore, in the present discussion we are focusing on the data dealing with the changes caused by TTX treatment.

The results of the present experiment demonstrate that intravesical treatment with TTX leads to profound changes in the immunohistochemical characteristics of CaMG-UBPN. Interestingly, it has been previously proven that, like TTX, RTX also considerably influences the chemical coding of CaMG-UBPN [27]. As was mentioned in the Introduction section, although these toxins have different modes of action, their primary therapeutic effect is associated with inhibiting the nociceptive (sensory) transmission. Nevertheless, they both also seem to change the sympathetic neurotransmission. What is even more surprising, the present study has revealed that TTX intravesical instillation was followed by changes in the chemical coding of CaMG-UBPN very similar to those observed after RTX treatment in the previous experiment. In general, administration of RTX or TTX resulted in a significant decrease in the number of the CaMG noradrenergic neurons expressing NPY and VIP, and an increase in the number of noradrenergic neurons which stained for SOM, CB, GAL and nNOS [27]. Thus, it may be assumed that both these toxins, at least partially, exert their effect on the mammalian urinary bladder by influencing the sympathetic neurons. Moreover, taking into account a striking resemblance of the immunohistochemical changes found after treatment with either RTX or TTX, it can be assumed that this effect is accomplished by comparable mechanisms. Because it is well known that both RTX and TTX affect the sensory transmission, a direct influence of these toxins on CaMG neurons may possibly not be the case. In this context, the results obtained by Bossowska and Majewski [31,32] are particularly intriguing. They found that intravesical administration of RTX or TTX to pigs modifies the chemical coding of dorsal root ganglia (DRG) UBPN. These modifications at the DRG level may further affect CaMG neurons either through reorganization of autonomic projections at the spinal cord level or through afferent collaterals [33] which directly synapse on CaMG neurons.

In the present experiment no statistically significant differences were observed in the number and distribution of CaMG-UBPN between the control and TTX-treated pigs. This finding suggests that, at least in the case of the present study, the toxin did not evoke neuronal death. Generally TTX is considered as a factor causing the progressive degeneration of neurons, which are deprived of activity due to the chronic blockade of voltage-gated sodium channels [34,35]. Interestingly, contrary to this concept, Lysko et al. [36] have observed the neuroprotective effect of TTX on both cultured cerebellar neurons and on CA1 hippocampal neurons in gerbils exposed to brain ischemia. The lack of the neuronal loss found in the present experiment is possibly due to a relatively large distance between the place of TTX application (urinary bladder) and the investigated nerve cell bodies (CaMG). However, this finding may be considered as beneficial while taking into account the possible use of TTX as a potential drug for the treatment of diseases associated with the over-activity of the urinary bladder wall. In this context it is tempting to assume that CaMG-UBPN, through the release of NA, elicit contractions of the bladder base smooth muscle and relaxation of the bladder body (via β3-adrenoreceptors), thus leading to continence [37].

As was already mentioned, the present results clearly suggest that TTX, like RTX [27], is a factor evoking significant adaptation changes in CaMG-UBPN. It should be stressed that no morphological studies concerning the influence of TTX on the chemical coding of CaMG neurons innervating the mammalian urinary bladder have been performed so far. Nevertheless, it has been found by Burliński et al. [38,39,40] that in the female pig, intravesical application of TTX induces changes in the immunohistochemical characteristics of the paracervical ganglion (PCG) UBPN (PCG is considered to be a mixed autonomic ganglion consisting of both sympathetic and parasympathetic nerve cells; [41]).

Administration of TTX resulted in a slight but statistically significant decrease in the percentage of TH-immunopositive CaMG-UBPN. On the contrary, Burliński et al. [38] found that this neurotoxin induced an increase in the number of TH-immunoreactive neurons in the population of porcine PCG-UBPN. The dissimilarities regarding the noradrenergic neurons between both experiments may indicate that the noradrenergic nerve cells play different roles in urinary bladder control depending on their source.

Moreover, TTX administration caused distinct changes in the expression of most of the investigated biologically active substances in the FB+ noradrenergic neurons. Most probably, these substances function as neuromodulators or co-transmitters in relation to the main neurotransmitter NA. The changes observed after TTX treatment include a substantial decrease in the number of noradrenergic neurons expressing NPY and a statistically significant increase in noradrenergic neurons immunopositive to SOM, CB, GAL. TTX treatment was also followed by a decrease (absence) in the number of FB+ neurons revealing immunoreactivity to VIP. Moreover, some FB+ neurons immunopositive to nNOS appeared in the toxin-treated animals. However, it should be noticed that VIP or nNOS were expressed merely in single UBPN and thus the physiological relevance of changes concerning the expression of these substances is probably only, if anything, marginal.

The most spectacular modifications regarded the population of FB+/TH+/NPY+ UBPN, as their number was markedly reduced after TTX treatment. Tran et al. [42] found that in the rat lower urinary tract, NPY exerts a prejunctional inhibitory effect on the noradrenergic transmission. A similar observation was made by Ellis and Burnstock [43] who found that in the guinea pig, NPY neuromodulates co-transmission in the vas deferens by inhibiting the release of NA. These findings may suggest that the decrease in the NPY immunoreactivity after TTX treatment enhances NA release from the CaMG postganglionic fibers. As was mentioned previously, NA released from the sympathetic postganglionic terminals, by prompting contractions of the bladder base and relaxing the bladder body, leads to continence [37], so it can be assumed that TTX treatment may possibly promote continence.

Although TTX therapy, as was previously mentioned, also changed the expression of other neurotransmitters (or their markers) in the noradrenergic UBPN, it is difficult to discuss these data, since the function of the aforementioned neurotransmitters in correlation with NA in urinary tract tissues is not yet known. However, interestingly, Honda et al. [44] found that intrathecal administration of galanin in rats delays the onset of micturition, suggesting the inhibitory role of the galaninergic system in the control of the micturition reflex. It can be therefore supposed that the upregulation of GAL expression after TTX treatment may support the main NA function in the urinary bladder, which is achieving continence.

The present results have also revealed that TTX instillation resulted in some changes in the chemical coding of TH− (non-adrenergic) CaMG UBPN. Precisely, administration of the toxin was followed by a statistically significant increase in the number of FB+/TH− neurons expressing immunoreactivity to SOM, CB, GAL or nNOS, and a decrease in the number of nerve cells immunopositive to NPY or VIP. Therefore, it may be expected that the mechanism of TTX action is at least partly mediated by the non-adrenergic (thus possibly cholinergic or non-adrenergic, non-cholinergic) CaMG neurons.

In conclusion, the present study for the first time has provided some evidence that the intravesical administration of TTX induces profound changes in the chemical coding of the CaMG-UBPN.

## 4. Materials and Methods

### 4.1. Laboratory Animals

The study was performed on 12 juvenile (eight to 12 weeks old, 15–20 kg body weight, b.w.) female pigs of the Large White Polish race. The animals were kept under standard laboratory conditions. They were fed standard fodder (Grower Plus, Wipasz, Wadąg, Poland) and had free access to water.

Before performing any surgical procedure, all the pigs were pretreated with atropine (Polfa, Lublin, Poland, 0.04 mg/kg b.w., s.c.) and azaperone (Stresnil, Janssen Pharmaceutica, Beerse, Belgium, 0.5 mg/kg b.w., i.m.), and after 30 min the main anesthetic drug, sodium pentobarbital (Tiopental, 0.5 g per animal), was given intravenously in a slow, fractionated infusion. The depth of anesthesia was monitored by testing the corneal reflex.

### 4.2. Surgical Procedures

In all the pigs, a mid-line laparotomy was performed and the urinary bladder was gently exposed to administer a total volume of 40 μL of 5% aqueous solution of the fluorescent retrograde tracer Fast blue (FB; Dr. K. Illing KG & Co. GmbH, Gross Umstadt, Germany) into the wall of its right side in multiple injections. To avoid leakage, the needle was left in each place of FB injection for about one minute.

Three weeks later, which is an optimal time for the retrograde tracer to be transported to the CaMG and label urinary bladder-projecting neurons (UBPN) [45,46] the pigs were divided into two groups. Six pigs served as the controls and they were treated with intravesical instillation of citrate buffer (pH 4.9, 60 mL per animal). Another group of six pigs was treated with intravesical instillation of TTX (12 μg of TTX dissolved in 60 mL of citrate buffer pH 4.9). Ten minutes after the infusion, the contents of the bladder were evacuated and the catheter was removed.

One week after the administration of the citrate buffer or TTX, all the pigs were deeply anaesthetized with sodium pentobarbital and transcardially perfused with 4% buffered paraformaldehyde (pH 7.4). All CaMGs were postfixed in the same fixative (10 min at room temperature), washed several times in 0.1 M phosphate buffer and stored in 18% buffered sucrose at 40C until sectioning.

### 4.3. Sectioning of the Ganglia, Estimation of the Total Number of the CaMG-UBPN and Immunohistochemical Procedure

All details concerning sectioning of the ganglia, estimation of the total number of CaMG-UBPN and immunohistochemical procedure were entirely corresponding to those described in our previous contribution [27]. Immunohistochemistry involved double stainings which were performed according to an earlier described method [47]. They were applied to cryostat sections from both the ipsi- and contralateral CaMG. The sections were selected from three different representative regions of the ganglia (upper one-third, middle and lower one-third).

Immunohistochemical characteristics of FB+ neurons were investigated using primary antibodies against TH (marker of noradrenergic neurons-more precisely, TH is a marker of catecholamines, however, it is commonly accepted that the main catecholamine utilized by peripheral sympathetic neurons is NA, therefore the term noradrenergic will be used throughout the paper), NPY, VIP, SOM, CB, GAL and nNOS (details concerning all the primary and secondary antibodies used are listed in Table 3).

TH antiserum was applied in a mixture with antisera against NPY, VIP, SOM, CB, GAL or nNOS, respectively. The presence of the above mentioned active substances (NPY, VIP, SOM, CB, GAL) or their markers (TH, nNOS-enzymes of the catecholamine or nitric oxide biosynthesis pathway, respectively) was earlier revealed in CaMG neurons in the pig [27].

The application of antisera raised in different species allowed investigation of the coexistence of TH with other substances. Retrogradely and immunohistochemically labeled perikarya were evaluated under Olympus BX51 microscope equipped with epi-fluorescence and an appropriate filter set for CY3-conjugated streptavidin and fluorescein isothiocyanate (FITC).

To determine percentages of the particular neuronal subpopulations, at least 300 of FB+ neuronal profiles investigated with one combination of the primary antisera were counted in both (left and right) CaMG in each animal studied. The percentages of the retrogradely labeled neurons immunopositive to particular biologically active substances or their markers investigated were pooled in all the animals and presented as means ± SD.

Micrographs were taken using an Olympus XM10 digital camera. The microscope was equipped with cellSens Dimension Image Processing software. Morphometric data relative to each neuronal class were compared within each animal and among the animals, and were analyzed by the Student *t*-test (using GraphPad PRISM 4.0 software). The differences were considered significant at *p* < 0.05.

### 4.4. Control of Specificity of the Tracer Staining and Immunohistochemical Procedures

The details concerning control of specificity of the tracer staining were analogous to those described in our previous study [27]. All these procedures excluded any leakage of the tracer and validated the specificity of the tracing protocol.

To test the specificity of primary antibodies, preincubation tests were performed with the sections from the control pigs (their inactivation by excess amount of antigens was performed before using to stain the control sections; Table 4). Preincubation of 1 mL of the corresponding antibody at working dilution with 20 μg/mL of the respective peptide (overnight at 4 °C) completely abolished the immunoreaction. The omission and replacement of the respective primary antiserum with the corresponding non-immune serum also served as a negative control. After staining no immunoreaction was visualized.

## Figures and Tables

**Table 1:** Percentages of Fast blue-positive (FB+) noradrenergic neuronal subpopulations in the caudal mesenteric ganglia (CaMG) of the control pigs and in the animals after tetrodotoxin (TTX) treatment (TH tyrosine hydroxylase, NPY neuropeptide Y, VIP vasoactive intestinal polypeptide, SOM somatostatin, CB calbindin, GAL galanin, nNOS neuronal nitric oxide synthase). Data expressed as mean ± SD. Asterisks mark statistically significant differences at * *p* < 0.05, ** *p* < 0.001, *** *p* < 0.0001.

Experimental Group	FB+/TH+	FB+/TH+/NPY+	FB+/TH+/VIP+	FB+/TH+/SOM+	FB+/TH+/CB+	FB+/TH+/GAL+	FB+/TH+/nNOS+
Control pigs	92.6% ± 3.4%	88.9% ± 5.3%	1.7% ± 0.6%	8.8% ± 1.6%	1.8% ± 0.7%	1.7% ± 0.8%	0%
TTX-treated pigs	88.8% ± 2% *	10.6% ± 5.3% ***	0% ***	39% ± 12.8% ***	12.6% ± 2.7% ***	10.9% ± 2.6% ***	1.1% ± 0.3% **

**Table 2:** Percentages of Fast blue-positive (FB+) non-adrenergic neuronal subpopulations in the caudal mesenteric ganglia (CaMG) of the control pigs and in the animals after tetrodotoxin (TTX) treatment (TH tyrosine hydroxylase, NPY neuropeptide Y, VIP vasoactive intestinal polypeptide, SOM somatostatin, CB calbindin, GAL galanin, nNOS neuronal nitric oxide synthase). Data expressed as mean ± SD. Asterisks mark statistically significant differences at * *p* < 0.05, ** *p* < 0.001, *** *p* < 0.0001.

Experimental Group	FB+/TH−	FB+/TH−/NPY+	FB+/TH−/VIP+	FB+/TH−/SOM+	FB+/TH−/CB+	FB+/TH−/GAL+	FB+/TH−/nNOS+
Control pigs	7.4% ± 3.4%	27.3% ± 17.6%	8.9% ± 6.8%	0%	0%	0%	0%
TTX-treated pigs	11.2% ± 2% *	1.2% ± 2% **	0%	2.7% ± 1.5% **	1.9% ± 0.8% ***	3% ± 1.4% ***	4.2% ± 2.7% ***

**Table 3:** List of primary antisera and secondary reagents used in the study (TH tyrosine hydroxylase, NPY neuropeptide Y, VIP vasoactive intestinal polypeptide, SOM somatostatin, CB calbindin, GAL galanin, nNOS neuronal nitric oxide synthase, FITC fluorescein isothiocyanate).

Antigen	Code	Dilution	Host	Supplier
Primary antibodies				
TH	MAB318	1:400	Mouse	Millipore, Temecula, CA, USA
NPY	NA 1233	1:8000	Rabbit	Enzo Life Sciences; Farmingdale, NY, USA
VIP	VA 1285	1:4000	Rabbit	Enzo Life Sciences; Farmingdale, NY, USA
SOM	11180	1:4000	Rabbit	Icn-Cappel, Aurora, OH, USA
CB	Lot No.: 0.7	1:9000	Rabbit	Swant, Marly, Fribourg, Switzerland
GAL	AB 5909	1:1000	Rabbit	Millipore, Temecula, CA, USA
nNOS	AB 5380	1:6000	Rabbit	Millipore, Temecula, CA, USA
Secondary reagents				
Biotinylated anti-rabbit immunoglobulins	E 0432	1:1000	Goat	Dako, Germany
CY3-conjugated streptavidin	711-165-152	1:13,000	-	Jackson I.R.; West Grove, PA, USA
FITC-conjugated anti-mouse IgG	715-096-151	1:700	Donkey	Jackson I.R.; West Grove, PA, USA

**Table 4:** List of antigens used in pre-absorption test (TH tyrosine hydroxylase, NPY neuropeptide Y, VIP vasoactive intestinal polypeptide, SOM somatostatin, CB calbindin, GAL galanin, nNOS neuronal nitric oxide synthase).

Antigen	Code	Supplier
TH	AC21-0699-P	Abcore, Ramona, CA, USA
NPY	N3266	Sigma, St. Louis, MO, USA
VIP	V6130	Sigma, St. Louis, MO, USA
SOM	S9129	Sigma, St. Louis, MO, USA
CB	AC21-2748-P	Abcore, Ramona, CA, USA
GAL	G5773	Sigma, St. Louis, MO, USA
nNOS	N3033	Sigma, St. Louis, MO, USA
